# How Can We Not Waste Legacy Genomic Research Data?

**DOI:** 10.3389/fgene.2020.00446

**Published:** 2020-05-08

**Authors:** Susan E. Wallace, Emily Kirby, Bartha Maria Knoppers

**Affiliations:** ^1^Department of Health Sciences, University of Leicester, Leicester, United Kingdom; ^2^Centre of Genomics and Policy, McGill University, Montreal, QC, Canada

**Keywords:** consent, data sharing, policy, secondary research, genomic research

## Abstract

Enabling genomic and biomedical data to be shared for secondary research purposes is not always straightforward for existing “legacy” data sets. Researchers may not know whether their data meet ethical and regulatory requirements for sharing. As a result, these data, collected using public funds and the good will and efforts of the donors and investigators, may not be used beyond their original purpose. Single-use plastics are now being banned in many countries; single-use research should be avoided if possible. This paper describes a filter developed through the driver projects of the Global Alliance for Genomics and Health that can be used by researchers to help them determine the extent of sharing possible for their legacy data and actions to be taken to enable further sharing.

## Introduction

Sharing of research data between institutions and across national and international borders is an expectation for many involved in genomic research studies. Too often, though, datasets languish because, amongst other reasons, researchers are unaware of whether the original consent given includes further data sharing or whether existing ethical, legal, and institutional requirements allow such sharing. The Global Alliance for Genomics and Health (GA4GH) “…both advocates for responsible data sharing and produces the practical standards to enable such a future.” (Birney, 2019). Through its driver projects, “real-world genomic data initiatives” that help guide and implement data sharing activities^1^, and workstreams, stakeholders work together to develop policies, tools and standards that follow the GA4GH *Framework for Responsible Sharing of Genomic and Health-Related Data* which provides, within a human rights framework, “a set of foundational principles for responsible research conduct and oversight of research data systems in the realm of genomic and health-related data sharing.” (Knoppers, 2014). The recently revised GA4GH Consent Policy^2^, which was written, “…to guide the sharing of genomic and health-related data in a way that supports the autonomous decision-making of data subjects,” states that tools should be developed to support data donors^3^ ‘ understanding of data sharing plans and to ensure data are shared as was agreed in the consent. This filter is one example of a flexible tool that, as part of a larger governance framework, can help researchers determine if legacy datasets can be shared, within applicable ethical, and legal requirements while respecting patients and participants’ wishes.

## Materials and Methods

### Policy Background

**FIGURE 1:**
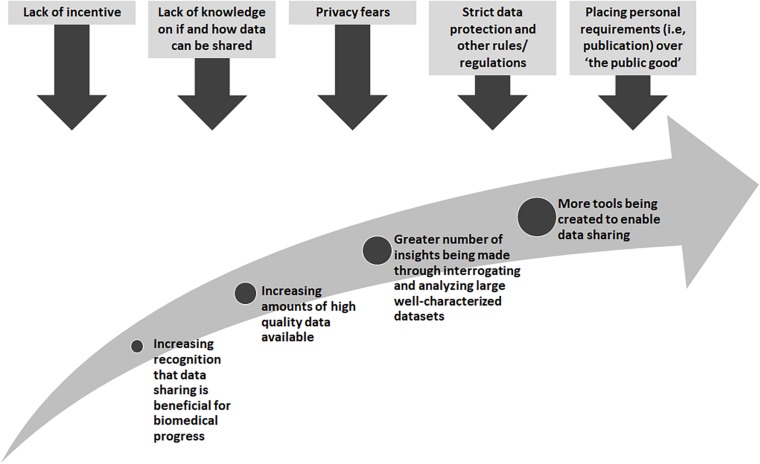
Incentives for and pressures against greater sharing of biomedical data.

There are many obstacles that stand in the way of wide-spread data sharing, yet there are also great incentives and rewards (Figure 1). Many existing “legacy” datasets from research or datasets generated from legacy or archival biological samples were created before widespread data sharing was encouraged. In these cases, research proposals and consent materials commonly did not include plans and language to enable further sharing, and often included conditions that limited the way in which a researcher could share, for example, across international borders or for research in other disease types than studied in the original research. At one time, sharing datasets could not be done easily so it was normal to not think about these possibilities. With technological changes and the genomics and big data revolutions, interrogating large datasets is now the norm, and in some cases, the only way in which the fundamental causes of disease could be found. Funders in many countries now require data sharing as part of grant conditions, and groups such as the GA4GH have worked tirelessly to develop tools and policies to share data for research purposes in a scientifically sound, ethical and lawful way. However, there are still barriers to overcome. The “…sharing of data and samples through global collaborative research networks…” has raised fears of a loss of privacy (Kaye, 2012). New legislation, such as the recent European General Data Protection Regulation (GDPR)^4^, has caused many to be unsure as to what can and cannot be done^5^. In addition, there are those who might feel they cannot share, for many reasons, such as a misplaced commitment to “protect the privacy” of their participants or the need for secrecy in order to be the one to publish that ground-breaking, and promotion-securing, academic paper (Linek et al., 2017).

For those who actively seek to share legacy data, by for example joining a national or international consortium, the options may be reduced to not sharing, in effect wasting the opportunity to achieve the best from the data collected, or sharing in a way that might not fully reflect the original wishes of the data donors. A stepwise approach to assessing legacy datasets would allow researchers to decide how they could share their data to the greatest extent possible. The, “…ethical and legal interoperability process…” for assessing retrospective or “legacy” studies, proposed by Tassé et al. (2016) was chosen as a framework. This process asks the researchers to (1) identify the legal and ethical restrictions inherent in a data set, (2) determine whether these would allow or prevent participation in research collaborations, and (3) identify any options that would help to resolve these in an ethical and lawful way. Two GA4GH driver projects have now taken these steps and used them to construct a “legacy filter” to determine ethical and legal interoperability. While the use of the filter is different for each of the driver projects described below, the approach for creating or tailoring a filter can be used by anyone seeking to identify the requirements for sharing and using legacy data. The expectation is that any filter would be used within any existing governance framework and would inform, not exclude, other measures such as the use of data access agreements (DAAs) and other appropriate safeguards.

### The International Cancer Genome Consortium (ICGC) and the Accelerated Research in Genomic Oncology Project (ICGC-ARGO)

The International Cancer Genome Consortium (ICGC) was established in 2008 to broadly and comprehensively map the structural aberrations of genomes and begin to understand the molecular basis of cancer (Hudson et al., 2010). Data are now available for over 88 projects across 17 jurisdictions (16 countries and the European Union) with >20,000 tumor genomes for 26 cancer types. The results of the analyses of these data are available through the Data Coordination Centre (DCC) via the ICGC website^6^. The ICGC Accelerated Research in Genomic Oncology (ARGO) project follows on from ICGC and, “…aims to analyze biospecimens from at least 100,000 cancer patients with high quality clinical data to address current key outstanding questions that are vital to our quest to defeat cancer.”^7^ This GA4GH driver project is an international research consortium of public studies and private commercial entities. Because research and clinical data from individuals will be contributed from many different countries with differing ethical, cultural and legal norms, ethical, and legal interoperability across studies is key.

Upon joining the consortium, ICGC members agreed to make the data as broadly available as possible under appropriate governance with minimal restrictions to expedite cancer and related research. It was recognized early that a core set of ethics elements were needed for researchers to include in consent materials given to, and in discussions with, prospective research participants. Two lists were created: a set of core elements member projects must agree to and a list of elements where there would be flexibility. For example, sharing with colleagues internationally was core, while decisions on whether to return individual research results were given over to the local member project to make. A later analysis of member study consent materials showed that, due to projects using their own institutionally approved consent materials in many different languages, it was very difficult to ascertain whether the core ICGC elements were being clearly communicated to participants (Wallace and Knoppers, 2011). While no concerns were raised (and to date this continues to be the case), anecdotal discussions highlighted that ambiguous consent language in ICGC member consents could preclude participation. For example, originally ICGC core elements stated that data would be used for cancer research but once requests for the data began to be received, it became clear that the ICGC data were useful for research related to, but not specifically for, cancer. It was decided that the scope of research in the core elements should be broadened to “cancer and related research” and later to “any approved biomedical research.” This raised the question of whether the member projects were still in compliance. A letter was sent to each project leader asking them to confirm if their project had consent for two key elements: broad research use and international data sharing. If the project leader could not answer yes to these, they were instructed to speak with their ethics committee to see if it was possible to re-consent their participants or obtain a waiver (if possible and appropriate under local legal and ethics requirements.) Throughout the project, all member project leaders have completed this form. At least one ICGC project re-consented its participants for the broader scope of research (Wallace and Knoppers, 2011).

For ICGC-ARGO, a set of core ethics elements was again agreed on. It was decided that it would be beneficial if the projects could confirm whether their consent adhered to the details of the consortium at the beginning of the process of becoming a member, rather than retrospectively seeking this confirmation as with ICGC. Using the filter process, the authors reviewed the thirteen elements that had been drafted for ICGC, and translated them into a limited number of process-related elements (six) that it was felt required confirmation; other elements would be followed up on through other processes. For example, one core element is that data users will attest that they will not attempt to re-identify participants. As this is a provision in the legally binding DAA used by ICGC-ARGO, it was felt that enforcing this should be governed through the data access process which requires institutions to take legal responsibility for the actions of its researchers.

**FIGURE 2:**
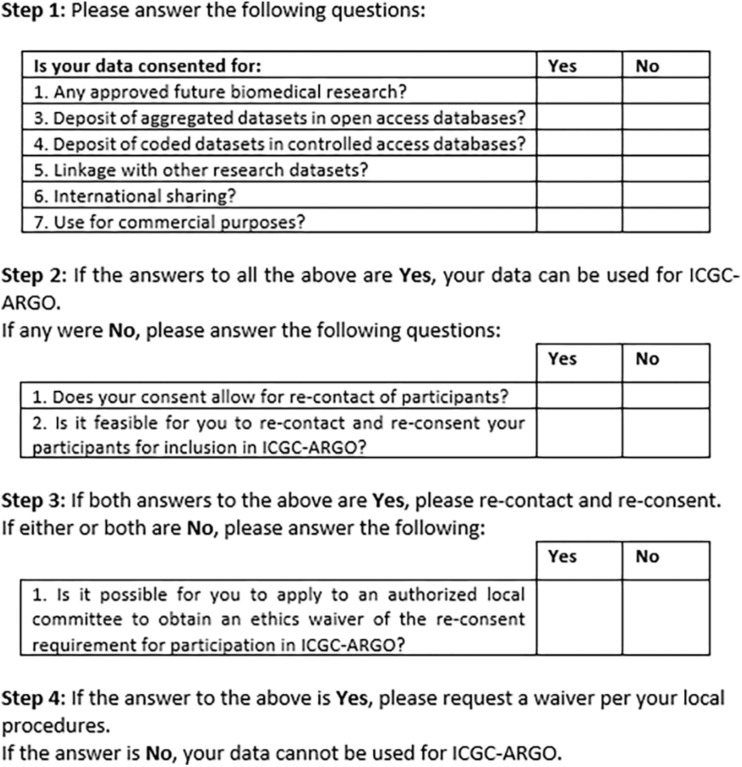
ICGC-ARGO consent assessment tool for participation.

Figure 2 shows an early version of the filter for use by ICGC-ARGO as part of its Expression of Interest (EOI) process. This shows the seven core research consent elements of participation in this consortium but also provides further steps that researchers can take to enable their dataset to be shared respecting research ethics requirements. Step 1 takes one through the six and if these points cannot be met, Step 2 asks if re-contact and re-consent are possible. If not, applying for a waiver from an appropriate body, such as a research ethics board, is suggested. This will be useful in cases when re-contact was not foreseen or for when it is may be impracticable to re-consent participants. Consent language would need to be interpreted to judge whether it fits with the items in the tool, so contact information of an ICGC-ARGO team member is available if guidance is needed. A possible further option would be to anonymize the dataset. This is not the preferred option as de-linked data would be of limited value in clinical care and may pre-empt projects from updating datasets longitudinally.

An early version of the retrospective assessment filter was developed and informally piloted with a small number of ICGC projects that were considering joining ICGC-ARGO and this showed that ambiguous consent text could preclude participation. For example, if there is research consent to share data with one other named country, outside the one in which the research is being conducted, can the consent be interpreted as allowing “international data sharing” with any country? The wording of point 6: “Use by industry partners” has been changed as a result of discussions with potential project representatives and the ARGO Ethics and Governance Committee – should it be specifically aimed at commercial entities or made broader, such as “Use by bona fide researchers from institutions including not-for profit and commercial?”

Those completing the ICGC-ARGO EOI application must mark their agreement to this statement: We agree that their participant (donor) consents meet the requirements of inclusion in ICGC-ARGO as outlined in the ICGC-ARGO Participant Assessment tool in Appendix III. By including the retrospective assessment filter tool at this point in the formation of the consortium, we have tried to place consent harmonization at the heart of the recruitment process, and not as an afterthought. However, in anecdotal discussions with project representatives it appears that boxes may have been ticked without a full understanding of the specific project’s informed consent materials. Discussions continue as to how to when and where would be the best place to introduce the filter. There are plans to automate this process by including a requirement to complete the filter as part of the online data submission process. This could allow greater scope for explaining the specific elements and for recording acceptance of its provisions.

### The Human Cell Atlas

A similar “legacy assessment filter” is currently being developed in the context of the Human Cell Atlas (HCA) driver project. Given the diversity of tissues types and cells required to map the human body, the HCA presents an interesting scenario, since several contributors to the Atlas will need to consider both the use of legacy tissue samples (for e.g., in the case of rare specimens, or tissues collected prior to the creation of the HCA), as well as legacy datasets. In this perspective, the first draft of the HCA research consent assessment filter was divided into four steps, and namely:

1. Can the legacy tissue sample be used to generate datasets for the HCA?
2. Is the tissue donor’s consent adequate to deposit datasets in the HCA data coordination platform?
3. What is the appropriate data tier for the datasets?
4. If requirements for previous steps are not met, is it possible to re-consent donors or seek an ethics consent waiver?

Steps 2 and 4 are similar to the elements used in the ICGC-ARGO filter. However, Step 1 was added in light of the complexities involved in the tissue sampling sources and scenarios envisaged by HCA contributors^8^. Furthermore, Step 3 was added to account for the potential levels of permission on data sharing for example, based on consent language, data protection requirements, source of tissue (e.g., paediatric, disease cohorts), or other policy requirements, such as open (public) versus registered versus managed access^9^.

Although at the time of writing, the assessment filter is still being discussed within the HCA Ethics Working Group, it is hoped that the final filter will provide an educational guidance tool, pointing to different layers of considerations involved in the use of legacy tissues samples and datasets. We expect that pure legal compliance will depend on more than simply this assessment tool (for example, on data protection regulation, institutional policies, and ethics approvals, etc.). Nonetheless, dissemination of this tool to the HCA community aims at fostering an understanding of transparent, and responsible data governance, while maximizing legacy data sharing and use.

## Discussion

When initially prepared, the main objective of the legacy filter was to provide guidance on assessing whether research consent language used by member projects was sufficient to allow sharing within consortia, in response to the authors’ experience with seeking interoperability between the ethical, legal and social issues (ELSI) linked to research studies. Because the teams organizing most scientific consortia are not “legal entities” they cannot enforce decisions across consortia, instead they must rely on each participating study to be able participate based on their own local legal requirements and cultural norms. Better harmonization of these, such as around sharing legacy data, would be beneficial to consortia, but has been shown that it would be difficult to achieve (Tassé et al., 2010). Data protection regulations, such as the GDPR, can add an additional layer of complexity to this reliance on local practices and knowledge. Consortia need to be aware that use of the filter does not in itself verify compliance. It is always contingent on the researcher being compliant with their own locally applicable data protection regulations. However, in their local adaptations of legacy filter tools, regional consortia may eventually consider adding additional steps to provide guidance on jurisdiction-specific data protection requirement (e.g., GDPR).

The filter can help with clarifying the consent elements needed for participation but familiarity with consent materials is needed. When principle investigators of a research study seek to be part of research consortia they may not have the in-depth knowledge of the ethical, social and legal requirements under which they must act, leaving the ELSI representatives (if there are such individuals) to raise concerns about whether participation conforms with the rules under which the data were gathered. Therefore, it is crucial that all researchers understand that they are taking responsibility for knowing, not only the content of their consent materials, but what their local (institutional or national) rules and regulations are, so that when they tick the boxes they do so in full knowledge of the commitment being made. Groups and individuals, such as data protection officers within institutions and research ethics committees, have a role in educating and working with their research teams, as well as learning themselves about working in national and international consortia.

## Actionable Recommendations

It is vital that research data is shared for purposes that adequately match the understanding and consent given by data donors and that conform with applicable ethical, social, and legal requirements. While it is well-known that individuals may not remember the exact provisions in any given consent form that they have signed, it is also known that one of the most important considerations underlying agreement to participate in research is that researchers and academic institutions are worthy of their trust (Dixon-Woods and Tarrant, 2009). In addition, neither of the filter examples presented have been in place long enough to critically evaluate their success. Empirical evidence will be needed to validate the approach taken.

Therefore, we recommend that:

1. International consortia agree on a set of core elements for participation, design a filter to reflect these to be provided to study leaders considering participation.
2. Project leads attest that their consent materials meet the requirements for participation and that this attestation be recorded either on paper or through electronic means.
3. Consideration be given to the best way to present the filter, such as through EOIs or data submission processes.
4. Consortia use this and similar tools to educate their communities and raise awareness with respect to the complexities involved in the ethical governance of legacy datasets.
5. Local data protection officers, research ethics committees and others, such as legal experts, work with researchers to educate them on the ethical, social, and legal requirements surrounding data sharing.
6. Consortia that have used the filter share their experiences in order to enable improvements to be recommended.

## Conclusion

This filter is proposed as one part of a larger governance framework to support research consortia. Its aim is not to place barriers in the way of researchers, but instead provide a way for them to know what contributing data to a consortium entails and to have a simple way to confirm that their consents meet the requirements for participation. If there are conditions that block participation, researchers will know what avenues they can take to share their data according to ethical and legal requirements. Datasets, like plastics, cannot continue to be single use. This filter is one way to encourage data sharing to the widest extent possible, in a responsible, ethical and lawful way that respects the wishes of the original data donor.

## Author Contributions

SW drafted the original draft of this manuscript. All authors contributed to the writing and editing of the text, and approved the final text.

## Conflict of Interest

The authors declare that the research was conducted in the absence of any commercial or financial relationships that could be construed as a potential conflict of interest.

## Footnotes

1 Global Alliance for Genomics and Health. Driver Projects. Available at https://www.ga4gh.org/how-we-work/driver-projects/. Accessed 30Mar20.

2 Global Alliance for Genomics and Health Consent Policy (Sept. 2019). Available at https://www.ga4gh.org/wp-content/uploads/GA4GH-Final-Revised-Consent-Policy_16Sept2019.pdf. Accessed 24Nov19.

3 The term “data donor” has been chosen to be used throughout and is defined in the GA4GH Lexicon as, “The individual whose data have been collected, held, used and shared.” Available at: https://www.ga4gh.org/wp-content/uploads/GA4GH_Data_Sharing_Lexicon_Mar15.pdf. Accessed 08Apr20.

4 REGULATION (EUhile) 2016/679 OF THE EUROPEAN PARLIAMENT AND OF THE COUNCIL of 27 April 2016. Available at https://eur-lex.europa.eu/legal-content/EN/TXT/PDF/?uri=CELEX:32016R0679&from=EN. Accessed 24Nov19.

5 The GA4GH has launched the GDPR & International Health Data Sharing Forum policy briefs to answer important questions in order to assuage concerns that might be blocking sharing. These are available at: https://www.ga4gh.org/genomic-data-toolkit/regulatory-ethics-toolkit/. Accessed 02Apr20.

6 ICGC Data Portal. Available at: https://dcc.icgc.org/. Accessed 24Nov19.

7 Accelerating Research in Genomic Oncology. Available at: https://icgc-argo.org/. Accessed 24Nov19.

8 Building the Human Cell Atlas: Issues with Tissues. (2019) Available at: http://www.genomicsandpolicy.org/Ressources/Issues-with-Tissues_2019.pdf. Accessed 24Nov19.

9 The GA4GH Lexicon (https://www.ga4gh.org/wp-content/uploads/GA4GH_Data_Sharing_Lexicon_Mar15.pdf) defines open access as “Making data available without restriction.” An example of managed access is registered access, defined in the Lexicon as, “A system of authentication and self-declaration prior to providing access to data.”
